# Reliability of genomic evaluation for egg quality traits in layers

**DOI:** 10.1186/s12863-020-0820-2

**Published:** 2020-02-11

**Authors:** David Picard Druet, Amandine Varenne, Florian Herry, Frédéric Hérault, Sophie Allais, Thierry Burlot, Pascale Le Roy

**Affiliations:** 1 PEGASE, INRAE, Agrocampus Ouest, 16 Le Clos, Saint-Gilles, 35590 France; 2 NOVOGEN, 5, rue des Compagnons, Plédran, 22960 France

**Keywords:** Laying hens, Egg quality, Genomic evaluation, Accuracy, Single step

## Abstract

**Background:**

Genomic evaluation, based on the use of thousands of genetic markers in addition to pedigree and phenotype information, has become the standard evaluation methodology in dairy cattle breeding programmes over the past several years. Despite the many differences between dairy cattle breeding and poultry breeding, genomic selection seems very promising for the avian sector, and studies are currently being conducted to optimize avian selection schemes. In this optimization perspective, one of the key parameters is to properly predict the accuracy of genomic evaluation in pure line layers.

**Results:**

It was observed that genomic evaluation, whether performed on males or females, always proved more accurate than genetic evaluation. The gain was higher when phenotypic information was narrowed, and an augmentation of the size of the reference population led to an increase in accuracy prediction with regard to genomic evaluation. By taking into account the increase of selection intensity and the decrease of the generation interval induced by genomic selection, the expected annual genetic gain would be higher with ancestry-based genomic evaluation of male candidates than with genetic evaluation based on collaterals. This advantage of genomic selection over genetic selection requires more detailed further study for female candidates.

**Conclusions:**

In conclusion, in the population studied, the genomic evaluation of egg quality traits of breeding birds at birth seems to be a promising strategy, at least for the selection of males.

## Background

Genomic evaluation, based on thousands of genetic markers in addition to pedigree and phenotype information [1], has become the standard evaluation methodology in dairy cattle breeding programmes over the past years. It has allowed for the improvement of the accuracy of estimated breeding values (EBV) of young bulls and, consequently, their much earlier utilization. Thereby, the generation interval as well as the phenotyping costs have been reduced because of the cessation of the progeny testing of bulls [2]. More recently, avian breeders have started to implement genomic selection in their selection schemes. Indeed, despite the many differences between dairy cattle breeding and poultry breeding, genomic selection is deemed very promising for the avian sector, especially for layer selection [3–5]. However, to optimize avian selection schemes, one of the key parameters is to properly predict the accuracy of genomic evaluation.

One of the most important factors directly affecting evaluation accuracy is the makeup of the reference population. From the very beginning, genomic evaluation implied that the size of the reference population should not be too small [1, 2, 6]. However, it has also been shown that increasing the size of the reference population does not directly improve evaluation accuracy [7, 8]. Indeed, the close relationship of the reference population with the candidate population is more critical than the size of the reference population. The evaluation becomes more accurate as candidate haplotypes become increasingly well represented in the reference population [7, 9–12]. Aside from the makeup of the reference population, the number of training generations to use is another important question. Indeed, it has been shown that evaluation accuracy is impacted by the number of training generations used [13, 14], depending on the heritability of the traits.

The present study assesses the relevance of genomic evaluation in comparison with genetic evaluation in order to predict the breeding values of selection candidates for egg quality traits in a pure line of layers. The main objective was to evaluate the expected genetic gain with respect to those traits in order to move from genetic to genomic evaluation.

## Results

Five egg quality traits related to eggshell quality and internal egg quality were studied: egg weight (EW), eggshell colour (ESC), eggshell strength (ESS), albumen height (AH) and eggshell shape index (ESshape).

Two phenotypic datasets were used. The first one, referred to as CC, contains data recorded from young hens (30 to 50 weeks old) bred in collectives cages. The second one, referred to as IC, contains data recorded from older hens (60 to 80 weeks old) bred in individual cages.

Three cases scenarios were studied. Case 1: evaluation at birth, Case 2: evaluation at 60 (CC) or 80 (IC) weeks of age, Case 3: evaluation at 140 (CC) or 160 (IC) weeks of age.

### Genetic parameters

Heritabilities remained steady whether REML was carried out with BLUP or with ssGBLUP and independently of the dataset under analysis, e.g., complete or partial (cases 1, 2 and 3). These results were observed whatever the trait or age (CC or IC). Differences ranged from 0% for ESshape in IC to 5% for EW in IC, and values were always higher with ssGBLUP than with BLUP (data not shown). Similar results were obtained for repeatabilites. Genetic correlations were even more stable than heritabilities or repeatabilities. Consequently, variance-covariance matrices were set for the rest of the study. The variance-covariance matrix obtained with genomic evaluation through the use of the complete dataset, i.e., REML carried out with the maximum amount of information, was used to perform subsequent BLUP and ssGBLUP.

Estimates of genetic parameters, obtained using ssGBLUP through the use of the complete dataset, are given in Table 1. EW, ESC and ESshape were highly heritable, while heritability (resp. repeatability) of ESS and AH were more moderate. For all traits, heritability was higher in CC than in IC and was of the same order of magnitude as repeatability in IC. This result may be due to an overestimation of additive genetic variance, which was partly confounded by common environmental effect in CC, as discussed before (see “Statistical model” section). Genetic correlation between EW and AH was positive and moderate, and showed no significant difference between CC and IC (resp. 0.37 and 0.35) (Table 1). Genetic correlations between ESshape and ESS, or between ESshape and AH, were also positive and moderate, but significantly higher in IC (resp. 0.28 and 0.20 in CC, and 0.45 and 0.41 in IC). Genetic correlation between AH and ESS was slighlty lower (0.12 in CC and 0.21 in IC). EW and ESshape were weakly correlated (0.12 in each dataset), while ESC was not correlated with other traits in CC but ESC was weakly correlated with ESS (0.16) and ESshape (-0.16) in IC.

**Table 1:** Genetic parameters for studied traits, estimated with ssGBLUP

Traits	EW	ESC	ESS	AH	ESshape
Collective Cages dataset					
EW	0.64 (0.02)	0.02 (0.02)	0.00 (0.02)	0.37 (0.02)	0.12 (0.02)
ESC	0.01 (0.01)	0.59 (0.02)	-0.02 (0.02)	0.03 (0.02)	-0.03 (0.02)
ESS	-0.02 (0.01)	0.05 (0.01)	0.27 (0.02)	0.12 (0.01)	0.28 (0.02)
AH	0.17 (0.01)	0.02 (0.01)	-0.00 (0.01)	0.34 (0.01)	0.20 (0.02)
ESshape	0.02 (0.01)	-0.06 (0.01)	0.18 (0.01)	0.12 (0.01)	0.48 (0.02)
Individual Cages dataset					
EW	0.45/0.69 (0.00)	0.02 (0.01)	0.14 (0.01)	0.35 (0.01)	0.12 (0.01)
ESC	0.02 (0.05)	0.40/0.57 (0.00)	0.16 (0.01)	0.08 (0.01)	-0.16 (0.01)
ESS	0.02 (0.05)	0.08 (0.03)	0.23/0.37 (0.03)	0.21 (0.00)	0.45 (0.01)
AH	0.10 (0.04)	0.02 (0.03)	0.01 (0.00)	0.26/0.36 (0.01)	0.41 (0.01)
ESshape	-0.03 (0.02)	-0.07 (0.01)	0.16 (0.01)	0.10 (0.02)	0.42/0.56 (0.00)

In diagonal: heritability/repeatability; upside diagonal: genetic correlations; downside diagonal: phenotypic correlations. Standard errors are in parentheses

Traits: EW: Egg Weight; ESC: Eggshell Colour; ESS: Eggshell Strength; AH: Albumen Height; ESshape: Eggshell shape index

### (G)EBVs relative accuracy for male candidates

#### CC traits

As expected, relative accuracy estimates (Additional file 1) significantly increased from case 1 to case 3 with the amount of phenotypic information available. In case 1, relative accuracy estimates were not homogeneous and varied depending on the trait. Accuracies for EW and ESshape tended to be low, with relative accuracy between 0.22 (EW genetic C2) and 0.24 (ESshape genetic C2), even though these traits were more heritable than ESS or AH, for which the relative accuracy was approximately 0.32 (AH genetic C2). These differences were less present in case 2 and no longer existed in case 3.

The results of the comparison between genetic evaluation and genomic evaluation are shown in Table 2, which gives the ratio of the relative accuracy of BLUP and ssGBLUP for each case studied. A value of 1 indicates no difference, values below 1 indicate that genomic evaluation is more accurate, while values above 1 indicate that genetic evaluation is more accurate.

**Table 2:** Ratio of relative accuracy from BLUP to ssGBLUP with respect to CC traits for candidates

Trait	Candidates	Case 1	Case 2	Case 3
EW	C1	1.05	1.05	1.05
	C2	0.75	0.96	-
	F	0.61	0.92	-
ESC	C1	1.39	1.00	1.01
	C2	0.78	0.92	-
	F	0.88	0.97	-
ESS	C1	0.87	0.89	1.01
	C2	0.99	0.90	-
	F	0.82	0.92	-
AH	C1	0.90	0.93	1.02
	C2	0.87	0.95	-
	F	0.77	0.88	-
ESshape	C1	0.73	0.87	1.01
	C2	0.56	0.82	-
	F	0.83	0.91	-

Traits: EW: Egg Weight; ESC: Eggshell Colour; ESS: Eggshell Strength; AH: Albumen Height; ESshape: Eggshell shape index

Candidates: C1: male candidates from G1; C2: male candidates from G1 and G2; F: female candidates from G2

Case 1: evaluation at birth; Case 2: evaluation at 60 weeks of age; Case 3: evaluation at 140 weeks of age

Evaluations carried out at birth (case 1) were the ones showing the greatest extent of difference between genetic evaluation and genomic evaluation. Results proved highly trait-dependent and ranged from 1.39 (ESC C1), e.g. a 39% gain in accuracy with genetic evaluation compared with genomic evaluation, to 0.56 (ESshape C2), e.g., a 44% gain in accuracy with genomic evaluation compared with genetic evaluation. There were strong disparities between C1 results, with a mean of 0.99, and C2 results, with a mean of 0.79. Overall, for C2, genomic evaluation allowed for greater accuracy, which can be explained by the size of the reference population. This advantage of genomic evaluation over genetic evaluation was also trait-dependent.

In the evaluations carried out with respect to ancestry and contemporary relatives (case 2), the differences between genetic evaluation and genomic evaluation were less significant, with values ranging from 1.05 (EW C1) to 0.82 (ESshape C2), and a global mean of 0.93. Differences between C1, with a mean of 0.95, and C2, with a mean of 0.91, were not as significant as they were in case 1, but still existed. As in case 1, the use of ssGBLUP allowed for a relative increase in accuracy.

Evaluations carried out in case 3 showed little difference between BLUP and ssGBLUP, with a mean close to 1.

#### IC traits

As was the case with CC, accuracy estimations were different for each trait (Additional file 2), depending on the evaluation scenario. The evolution of accuracy was also linked to the amount of phenotypic information available.

Here again, the increase in relative accuracy with genomic evaluation, compared to genetic evaluation, was observed in case 1 and in case 2 (Table 3). This increase was more significant for IC traits than it was for CC traits, both in case 1 (mean = 0.82) and case 2 (mean = 0.84). The global gain in accuracy observed in genomic evaluations carried out on C2, in comparison to those carried out on C1, was similar to the gain noticed for CC traits.

**Table 3:** Ratio of relative accuracy from BLUP to ssGBLUP with respect to IC traits for candidates

Trait	Candidates	Case 1	Case 2	Case 3
EW	C1	0.85	0.89	1.01
	C2	0.65	0.78	-
	F	0.77	1.01	-
ESC	C1	0.84	0.99	1.01
	C2	0.72	0.65	-
	F	0.85	1.01	-
ESS	C1	0.98	0.90	1.01
	C2	0.95	0.80	-
	F	0.69	1.01	-
AH	C1	0.87	0.88	1.01
	C2	0.73	0.77	-
	F	0.79	1.00	-
ESshape	C1	0.70	0.88	1.00
	C2	0.95	0.88	-
	F	0.83	1.01	-

Traits: EW: Egg Weight; ESC: Eggshell Colour; ESS: Eggshell Strength; AH: Albumen Height; ESshape: Eggshell shape index

Candidates: C1: male candidates from G1; C2: male candidates from G1 and G2; F: female candidates from G2

Case 1: evaluation at birth; Case 2: evaluation at 80 weeks of age; Case 3: evaluation at 160 weeks of age

Evaluations carried out in case 3 showed no differences between BLUP and ssGBLUP, with a mean very close to 1, as was the case for CC.

### (G)EBVs biases and dispersion for male candidates

The bias statistics exhibit an expected value of 0 if evaluation is unbiased. In both CC and IC (Additional files 3 and 4), biases were low and most often negative, indicating an underestimation of (G)EBVs when using partial datasets. The biases increased as the amount of phenotypic information decreased, from approximately 0 in case 3 to -0.11 in case 1. Biases were slightly higher with genomic values, compared to genetic values, in any given trait situation. The differences between traits or between candidate population C1 and candidate population C2 varied, without any clear tendency observed.

Unbiased estimators are supposed to have a regression slope equal to 1. Such unbiased estimators were observed in case 3, with regression coefficients estimated between 0.94 and 1.00 in CC and between 0.97 and 1.04 in IC (R-square values were approximately 0.98). This was the case for both genetic evaluation and genomic evaluation (Additional files 5 and 6).

In both CC and IC, the slopes decreased below 1 every time the amount of phenotypic information decreased. There was no significant difference between genetic evaluation and genomic evaluation: if slopes were closer to 1 when using genetic evaluation on CC traits, it was quite the opposite in the case of IC traits. Conversely, dispersion appeared to be significantly higher in CC than in IC: in the case of IC, slopes remained above 0.7, with few exceptions, even in case 1, while they often decreased below 0.7 in the case of CC (R-square values were between 0.25 and 0.30 in case 1, and between 0.40 and 0.50 in case 2). The slopes were also strongly linked to the evaluated traits, regardless of the candidate population.

### (G)EBVs relative accuracy for female candidates

#### CC traits

As was the case for males, accuracy estimations of (G)EBVs for females were not homogeneous, depending on the trait (Additional file 1): some traits were evaluated with greater accuracy than others, and accuracy evolution was not the same for all traits, depending on the scenario. However, these differences were not the same as those noticed with males. This could be a consequence of different genetic determinism of traits in females, for example, the impact of Quantitative Trait Loci carried by sexual chromosomes (females are ZW, and males are ZZ). Relative accuracy of (G)EBVs was nonetheless generally higher for females in comparison with males, especially in case 2 where females had their performances taken into account.

Furthermore, genomic values were always more accurate than genetic values and, as with males, the gain increased when the amount of phenotypic information was low (Table 2). Indeed, evaluations carried out at birth (case 1), showed a significant increase in accuracy with ssGBLUP evaluation compared to BLUP evaluation. The mean of ratios was close to 0.78, i.e. a 22% gain in accuracy with genomic evaluation compared with genetic evaluation. Regarding evaluations carried out in case 2, where the performances of the females were taken into account, this value was only 0.92, i.e., an 8% gain in accuracy.

#### IC traits

As was the case for CC, correlations were moderate (Additional file 2) and varied depending on the trait in case 1, while they were always very high (with a minimum of 0.93) in case 2, where the performances of the females were taken into consideration.

In case 1, the increase in accuracy noticed with ssGBLUP evaluation in comparison with BLUP evaluation (Table 3) was of the same order of magnitude as for CC, with a mean of ratios close to 0.79. In case 2, this value was between 1.00 and 1.01, depending on the trait.

### (G)EBVs biases and dispersion for female candidates

As was the case for males, and both in CC and IC (Additional files 3 and 4), biases were low and often negative (with an exception in case 1 EW and ESC). This indicated an underestimation of (G)EBVs when evaluating partial datasets. A similar increase in biases was observed for females when the amount of phenotypic information decreased. Here again, no clear relationship could be noticed between traits and biases, nor between the type of evaluation carried out (genetic or genomic) and biases, contrary to what was observed with males.

Regarding the regression coefficients in both CC and IC (Additional file 5 and 6: Tables), the results were similar to those observed using male candidates (R-square values were between 0.25 and 0.30 in case 1 and approximately 0.95 in case 2). Except for ESshape in case 1, the regression coefficients decreased below 1 every time the amount of phenotypic information decreased, i.e., results in case 2 were less biased than those in case 1.

As was the case for males, there was no significant difference between genetic evaluation and genomic evaluation, and dispersion seemed higher for CC than for IC. Once again, slopes were strongly linked to the trait evaluated in any given case.

## Discussion

### Genetic parameters

Estimates of heritability, repeatability and genetic correlations (Table 1) were in accordance with the literature [4, 15]. Moderate to high heritability coefficients enable significant expected genetic gains through selection with respect to the five egg quality traits. Selection carried out in order to increase eggshell strength should lead to an increase in albumen height, which is favourable to a combined selection of these traits. Increasing eggshell strength should also lead to an increase in egg short length at a given weight, which is unfavourable: e.g., the egg would be less ovoid. Finally, genetic correlations of eggshell colour to other traits are negligible, with the exception of eggshell strength in IC.

### Relevance of genomic evaluation for male candidates

In case 1 and in case 2, the results generally highlighted a greater accuracy of the evaluation of male candidates with ssGBLUP than with BLUP, in any given scenario, and particularly regarding IC traits. The difference between CC results and IC results can be explained by the nature of the data: indeed, IC data, referring to the hen itself and not just to the full sisters, allowed for the construction of a more accurate evaluation model. Otherwise, the results obtained in case 3 showed that the information about the grand-daughters had little impact on the evaluation. The fact of not using the performances of the grand-daughters does not seem to have any direct negative impact on evaluation accuracy.

The results observed in scenarios using C2 candidates tend to confirm those obtained using C1 candidates, and sometimes even amplified them. The difference between C1 results and C2 results could be explained by the increase in the size of the reference population, which went from 3 batches for C1 to 5 batches for C2, still with the same number of candidates. This increase in the size of the reference population had an impact on evaluation, with more candidate haplotypes represented in the reference population, as shown by Rabier et al. [9].

### Relevance of genomic evaluation for female candidates

The differences between traits, and the relationship between phenotypic information and accuracy noticed with males were also observed with females. Genomic evaluation provided more accurate evaluations than genetic evaluation, except for case 2, where IC traits were used. GEBV accuracy was capped at approximately 0.70 in CC, while it approached 1 in IC. This difference was because in CC, phenotypes are not related to a single bird, but to a cage of full sisters, which means that instead of being performed on the female, the evaluation was performed on its family.

Evaluations performed on a candidate population of females in IC (case 2) showed the relative accuracy of (G)EBV, which came close to 1. This result was because the performances of the females were taken into account for the evaluation: the addition of genomic information did not increase the gain in accuracy. Furthermore, as opposed to male candidates, females had very few daughters with performances: the lack of information about the performances of the daughters had little impact on the estimation of the value of the females.

### Towards the use of genomic evaluation of egg quality traits

In both CC and IC, the reliability of genetic evaluation or of genomic evaluation varied greatly depending on the trait. This heterogeneity in the impact on the evaluation can be explained by the differences found in the genetic architecture of the traits [16]. Indeed, when a trait is influenced by few QTLs with large effects, it would be poorly predicted with ssGBLUP, which assumes a common variance for all SNP effects. Moreover, a trait may be more or less influenced by non-additive effects, i.e., dominance and epistasis, which were not taken into account in the present study.

With regard to the comparison between genomic evaluation and genetic evaluation, with the same amount of phenotypic information used, genomic evaluation proved more accurate than genetic evaluation most of the time. As expected, the increase in accuracy of genomic evaluation was greater when phenotypic information was restricted.

Regarding the size of the reference population, it was observed that adding a generation, from C1 to C2, had an effect on the evaluation, as Weng et al. (2016) [13] showed. Augmentation of the reference population from 1 to 2 generations increased evaluation accuracy, especially when there was little phenotypic information available.

These results are very interesting for the poultry industry. Indeed, genomic evaluation and selection of males at birth would allow for their use at their sexual maturity, i.e., 6 months of age. For CC traits, there would be a significant loss in evaluation accuracy, although the loss would remain acceptable for IC. Depending on the weight of each trait in the breeding goal, this strategy would allow for a significant genetic gain for the global objective through an increment in selection pressure, i.e., it would be feasible to increase the number of male candidates for selection from 200 to 2000 and to reduce the generation interval from 18 months to 6 months.

The genomic evaluation and selection of females at birth would also allow for their use at their sexual maturity, i.e., 6 months of age. However, our results highlighted the fact that, for females, switching from selection at 18 months of age to selection at birth would result in a significant loss in evaluation accuracy. Moreover, in the case of females, selection pressure should not be significantly increased.

## Conclusions

Our results indicate that it seems advantageous to move from genetic selection at 18 months of age to genomic selection at birth (2 weeks in practice: the time to obtain genotypes and to calculate GEBVs), at least with respect to egg quality traits and as far as males are concerned. This strategy must be studied in greater detail for females in order to assess whether the implementation of genomic evaluation at birth would be an interesting option.

## Methods

### Animals

**Fig. 1:**
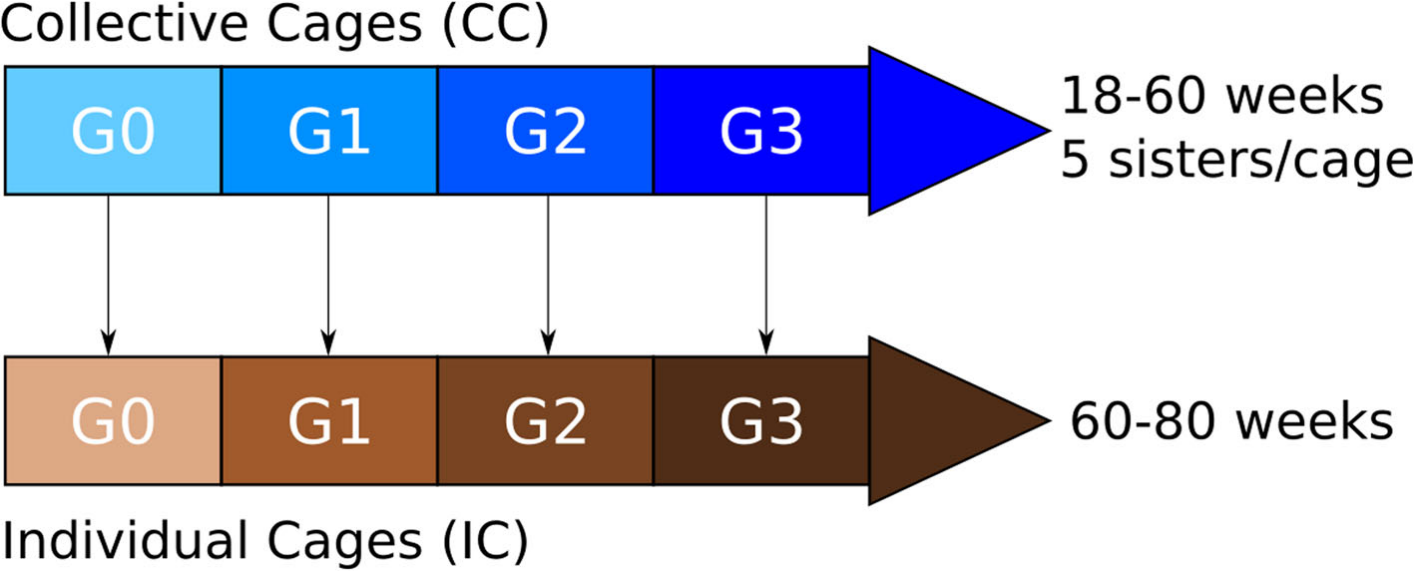
Population structure

For the purpose of this study, we have used the data of a pure line of Rhode Island layers selected by the breeding company Novogen (Plédran, France). The hens were hatched in 12 batches, born between 2008 and 2015, which corresponds to four generations (G0 to G3, Fig. 1), with three successive hatches per generation spaced 30 weeks apart. The genealogical information of all of the birds was recorded in the pedigree file, which concerned 2273 breeders: 514 sires and 1759 dams.

In each hatch, chicks were bred in a brooding area until the age of 18 weeks, and then transferred to collective cages of five full sisters for females (2997 collective cages) and to individual cages for males (200 males out of 2000 chicks were kept for the selection). Egg quality was recorded twice during this period, at 30 and 50 weeks of age. This step allowed for the makeup of our first phenotypic dataset, referred to as CC for collective cages, with a total of 14985 hens and 27915 eggs measured.

Then, at 60 weeks of age, a genetic evaluation was performed as a first selection, and 150 males and 650 hens were transferred to individual cages until the end of their careers at the age of 80 weeks. Egg quality was measured on a weekly basis. A total of 7982 hens, with 74976 performances, were concerned. This step allowed for the makeup of our second phenotypic dataset, referred to as IC for individual cages.

At the end of their careers, animals were slaughtered in poultry slaughterhouse.

### Genotypes

In this population, 2374 birds were genotyped using the 600K Affymetrix^®^ Axiom^®^ HD genotyping array [17]. Blood samples were collected from the brachial veins of the animals and DNA was extracted. For the first two generations, all male candidates were genotyped by Ark-Genomics (Edinburgh, UK) during the research project UtopIGe [16]. From the G2 generation onward, male and female reproducers were genotyped at the high-throughput genotyping platform Gentyane (Clermont-Ferrand, France).

Each animal was genotyped for 580961 SNP markers. According to the fifth annotation release of the *Gallus gallus* genome [18], these SNPs were distributed over macro-chromosomes (1 to 5), intermediate chromosomes (6 to 10), micro-chromosomes (11 to 28 and 33), one linkage group (LGE64), two sexual chromosomes Z and W, and a group of 3724 SNPs with unknown locations.

Genotypes were filtered through four successive steps: individuals with a call rate <95% were removed (0 animals excluded), SNPs with a MAF <0.05 were removed (258772 SNPs), SNPs with a call rate <95% were removed (7549 SNPs), and SNPs whose genotype frequencies deviated significantly from the Hardy-Weinberg equilibrium (P <10^−4^) were removed (12538 SNPs). Animals showing pedigree incompatibilities were also removed (12 individuals excluded). Thus, 302102 SNPs and a total of 1214 genotyped males and 1148 genotyped females were retained for the study.

### Traits

In this paper, traits are named according to Animal Trait Ontology for Livestock [19]. Five egg quality traits related to eggshell quality and internal egg quality were studied: egg weight (EW), eggshell colour (ESC), eggshell strength (ESS), albumen height (AH) and eggshell shape index (ESshape). Summary statistics of the traits are provided in Table 4.

**Table 4:** Summary statistics on phenotypic data

Traits	EW (*g*)	ESC (*withoutunits*)	ESS (*N*∗100)	AH (*mm*)	ESshape (*mm*.*g*^−1^)
Collective Cages dataset					
Number of records	27915	27932	25578	26447	25538
Mean	59.9	81.2	3785.3	5.5	1.1
Standard deviation	4.56	9.12	691.50	1.24	0.02
Min	41.00	35.36	870.00	1.10	1.04
Max	82.00	109.98	6620.00	11.70	1.17
Individual Cages dataset					
Number of records	74976	73033	65890	72107	67308
Mean	60.0	78.4	3671.7	4.7	1.1
Standard deviation	4.79	9.82	741.73	1.28	0.02
Min	41.70	30.55	682.00	0.90	1.02
Max	82.80	109.29	7187.00	9.00	1.17

Traits: EW: Egg Weight; ESC: Eggshell Colour; ESS: Eggshell Strength; AH: Albumen Height; ESshape: Eggshell shape index

#### Trait measurements

At 30 and 50 weeks of age for CC, and once a week for IC, the eggs produced on the farm were collected and quality traits were measured by the company Zootests (Ploufragan, France). The first step consisted in measuring egg short length (SLE in mm) and EW (in g), before calculating ESshape as follows: $ESshape = \frac {SLE/10}{(EW/10)^{1/3}}$. Then, eggshell colour was measured using a Minolta chromameter (Nieuwegein, The Netherlands) and three traits were recorded: redness of eggshell a*, yellowness of eggshell b* and lightness of eggshell L*. Eggshell colour was then calculated as follows: *ESC*=100−(*L*^∗^−*a*^∗^−*b*^∗^). Third, shell strength was measured using a compression instrument to evaluate the static stiffness of the shell. Eggs were compressed between two flat plates moving at constant speed. ESS is the maximum force recorded before eggshell fracture (in N, multiplied by 100). Finally, the egg was broken and AH (in mm) was measured using a tripod.

Equations for ESC and ESshape were those used by Novogen for egg quality control.

#### Choice of environmental effects to introduce into the model and elimination of outliers

For each trait, egg measurements were adjusted for environmental effects using the SAS ^*®*;^ 9.4 GLM procedure. The objective was to check which environmental conditions had significant effects on the traits and to remove outliers. The environmental effects tested were the hatch, the cage location in the poultry house during hatching, i.e., the battery and the cage location along the battery, the waiting time between sampling and egg measurement and the age of the hen.

All of these environmental effects were below the significance level (*P*<0.05), which means that they could be retained in the model of analysis. Raw data were then adjusted using the estimates of all of these effects, and outliers were deleted. The values presenting a deviation from the sire family mean of greater than 5 phenotypic standard deviations (0.4% of the CC performances and 0.01% of the IC performances) were considered as outliers.

Ultimately, a total of more than 25500 records for CC and more than 65800 records for IC were retained (Table 4). All distributions of adjusted phenotypes were symmetrical (data not shown).

### Genetic and genomic evaluations

Performances were centered (by subtracting the general mean) and standardized (by dividing by the standard deviation) before evaluation. Multi-trait evaluations were performed for the five traits using BLUP (EBV) and single-step GBLUP (GEBV) methodologies [20, 21]. BLUP was used as the reference method for genetic evaluations. ssGBLUP was used because a majority of our phenotypes came from non-genotyped birds (13837 hens were phenotyped, but not genotyped, of 14985 total hens), and this method allows those data to be accounted for in our evaluation. Before EBVs and GEBVs estimation, variance-covariance matrices were estimated using REML for both BLUP and ssGBLUP.

To perform those evaluations, the BLUPF90 family of programmes [22] was used. Variance-covariance matrices were first estimated using REMLF90 to obtain a good starting point for AIREMLF90. AIREMLF90 was then used to validate these variance-covariance components (the components have changed little between the two programmes) and estimate the standard errors of genetic parameter estimates. EBVs and GEBVs were then estimated using BLUPF90 [22].

#### Statistical model

The statistical model was the same for all of the traits and took into account the fixed and covariable environmental effects described before, plus the random genetic effects of the animal. For CC evaluations, each egg measured was associated with one cage of five full sisters, without knowing which hen laid which egg. As the measurements were repeated twice in CC, each hen had the expectation of two measured eggs, but it was not possible to take into account the common environmental effects of the hen. An “egg-animal” model, without common environmental effects, provided slightly more accurate (G)EBV than a sire model (data not shown). The inclusion of a “cage” random effect was also tested (instead of the fixed effects, which described the geolocation in the poultry building). The estimation of this variance-covariance component was very low, but the computation time was significantly higher with this model. The “egg-animal” model was then retained, and the heritability estimation was calculated as the ratio between the animal variance and the sum of animal and residual variances. Conversely, for IC evaluations, several measurements were available for each hen, and random common environmental effects of the hen were taken into account in the model. The heritability coefficient was therefore estimated as the ratio between the animal variance and the sum of animal, common environmental and residual variances, and the repeatability coefficient was considered as the ratio between the sum of animal and common environmental variances and the sum of animal, common environmental and residual variances.

#### Candidate populations

To assess the relevance of genetic evaluation and of genomic evaluation, the statistical properties (described below) of estimated breeding values, EBVs and GEBVs, of selection candidates were used.

**Fig. 2:**
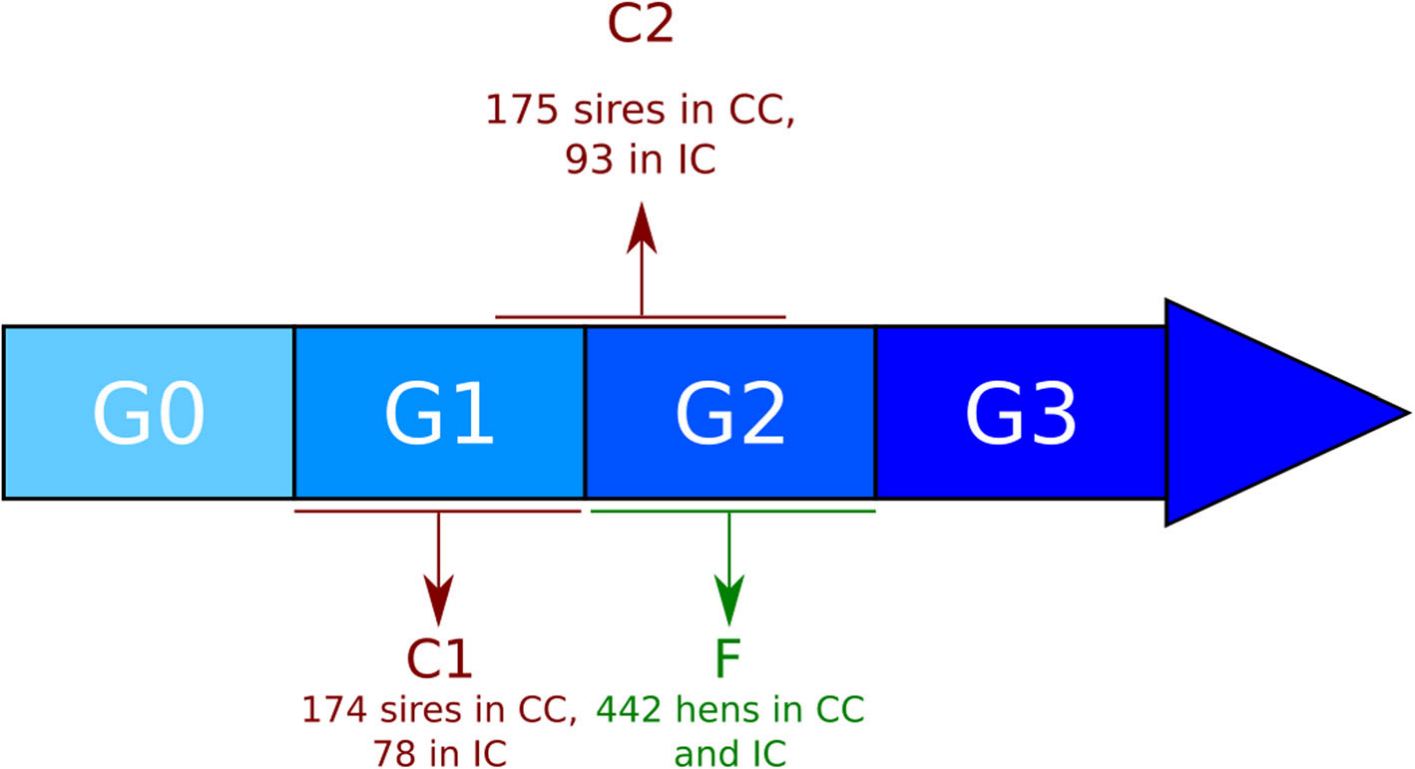
Candidate populations

A male candidate must have a genotype and have progeny tested. Two male candidate populations were considered: C1 and C2 (Fig. 2). In both, the number of sires in IC was smaller than in CC. This is due to the selection being carried out before moving them from CC to IC and to the fact that in IC, only sires having at least 8 daughters with performances were used.

The first male candidate population (C1) comprised birds from generation G1 that had daughters (G2) and grand-daughters (G3) with performances. This group was made up of 174 sires in CC and 78 sires in IC. A second male candidate population (C2) was considered in order to increase the reference population, therefore changing from three hatches for C1 to five hatches for C2, i.e., it comprised birds from the last hatch of G1 and the two first hatches of G2 (which was the limit to have male progeny with performances), which led to a total of 175 sires in CC and 93 in IC.

In addition to these male populations, a female candidate population (F) was formed using genotyped hens from G2 (Fig. 2). This group comprised 442 females in CC which were then moved to IC. The difference between this population and the male population was that IC females had performances available for evaluation and had few daughters (between 0 and 19 daughters/hen; 1.6 daughters/hen on average). As opposed to males, females were genotyped starting from G2, which means that it was not possible to have two female candidate populations.

#### Reliability of prediction

To assess the reliability of genetic evaluation and genomic evaluation, the estimated breeding values, EBVs and GEBVs, had to be compared to the true breeding values (TBVs) of candidates. However, TBV is never known when working with a real dataset. Moreover, it could not be approximated using Daughter Yield Deviation (DYD) [23], since our candidates had very few offspring. Therefore, to estimate the accuracy and bias of prediction of our evaluations, the LR method [24] was used.

The LR method uses correlation between complete and partial datasets to estimate the accuracy, since the amount of change expected in consecutive genetic evaluations was described as a function of their respective accuracies [25]. This method completes cross-validation approaches with semiparametric elements, based on the mixed model equations, to estimate the “population” accuracy. Population accuracy is relevant to compare the predictive ability of models and to maximize genetic progress.

The LR method relies on three statistics to estimate the accuracy and biases of an evaluation:

- The correlation between (G)EBVs from complete and partial evaluations to estimate relative accuracy
- The difference of means between (G)EBVs from complete and partial evaluations to estimate biases
- The slope of the linear regression of (G)EBVs from complete evaluation on (G)EBVs from partial evaluation to estimate the over- or underdispersion of estimates

To compare genetic evaluation and genomic evaluation using the same amount of data, a fourth statistic was used: the ratio between the relative accuracy of EBVs and the relative accuracy of GEBVs. This statistic allows quantification of the increase in accuracy expected when moving from genetic evaluation to genomic evaluation.

#### Application to data

All available pedigrees, phenotypes, and genotypes for GEBV estimation, from G0 to G3, constitute the complete dataset. Two cases of partial datasets were studied based on the amount of phenotypic information available when the evaluation was carried out:

- Case 1: The evaluation was carried out at birth of the candidates without considering the performances of their contemporary relatives nor the performances of the candidates, in the case of females. The evaluated population was limited to the candidates and their ancestors: all individuals had pedigree information; female ancestors had phenotypes; male ancestors and male (or female) candidates had genotypes for GEBV estimation.
- Case 2: The evaluation was carried out at 60 weeks of age for CC and at 80 weeks of age for IC, without considering the performances of the progeny of candidates. This case corresponds to the scheme classically used in layer selection. The evaluated population included the candidates, their contemporary relatives and their ancestors: all individuals had pedigree information; female ancestors, female contemporary relatives or candidates had phenotypes; male ancestors had genotypes for GEBV estimation; candidates, male or female, had genotypes for GEBV estimation.

Moreover, for C1 male candidates, the potential gain was also assessed taking into account the performances of their grand-daughters. In that case, the pedigrees and phenotypes of G3 hens were removed from the complete dataset to obtain a partial dataset (case 3).

The differences in significativity between relative accuracies, e.g., correlations as defined above, were assessed using the Hotelling-Williams test [26]. This test is used to compare two dependent correlations that share a common variable. The null hypothesis means that the two compared correlations are equal. The test statistics under the null hypothesis follow the Student’s t-distribution at n-3 degrees of freedom, with n being the number of observations. Observed correlations were compared two-by-two for EBVs and GEBVs at a significance threshold of 5%.

In layers, candidates are compared within a hatch: comparisons are never made between individuals from different generations for the purposes of selection. However, biases and dispersion statistics were presented here to illustrate the evolution of quality of evaluation according to cases 1 to 3.

## Supplementary information

**Additional file 1** Estimates of (G)EBVs relative accuracy in candidate populations with respect to cC traits. Traits: EW: Egg Weight; ESC: Eggshell Colour; ESS: Eggshell Strength; AH: Albumen Height; ESshape: Eggshell shape index. Candidates: C1: male candidates from G1; C2: male candidates from G1 and G2; F: female candidates from G2. Multi-trait evaluations were performed for the five traits using BLUP (EBV) and ssGBLUP (GEBV) methodologies. Case 1: evaluation at birth; Case 2: evaluation at 60 weeks of age; Case 3: evaluation at 140 weeks of age. In parentheses are groups determined by the Hotelling-Williams test at a confidence level of 95%. Groups a, b and c are used when EBVs correlations are compared to other EBVs correlations; d, e, f are used when GEBVs correlations are compared to other GEBVs.

**Additional file 2** Estimates of (G)EBVs relative accuracy in candidate populations with respect to iC traits. Traits: EW: Egg Weight; ESC: Eggshell Colour; ESS: Eggshell Strength; AH: Albumen Height; ESshape: Eggshell shape index. Candidates: C1: male candidates from G1; C2: male candidates from G1 and G2; F: female candidates from G2. Multi-trait evaluations were performed for the five traits using BLUP (EBV) and ssGBLUP (GEBV) methodologies. Case 1: evaluation at birth; Case 2: evaluation at 80 weeks of age; Case 3: evaluation at 160 weeks of age. In parentheses are groups determined by the Hotelling-Williams test at a confidence level of 95%. Groups a, b and c are used when EBVs correlations are compared to other EBVs correlations; d, e, f are used when GEBVs correlations are compared to other GEBVs.

**Additional file 3** Bias of (G)EBVs in candidate populations with respect to cC traits. Traits: EW: Egg Weight; ESC: Eggshell Colour; ESS: Eggshell Strength; AH: Albumen Height; ESshape: Eggshell shape index. Candidates: C1: male candidates from G1; C2: male candidates from G1 and G2; F: female candidates from G2. Multi-trait evaluations were performed for the five traits using BLUP (EBV) and ssGBLUP (GEBV) methodologies. Case 1: evaluation at birth; Case 2: evaluation at 60 weeks of age; Case 3: evaluation at 140 weeks of age

**Additional file 4** Bias of (G)EBVs in candidate populations with respect to iC traits. Traits: EW: Egg Weight; ESC: Eggshell Colour; ESS: Eggshell Strength; AH: Albumen Height; ESshape: Eggshell shape index. Candidates: C1: male candidates from G1; C2: male candidates from G1 and G2; F: female candidates from G2. Multi-trait evaluations were performed for the five traits using BLUP (EBV) and ssGBLUP (GEBV) methodologies. Case 1: evaluation at birth; Case 2: evaluation at 80 weeks of age; Case 3: evaluation at 160 weeks of age

**Additional file 5** Slopes of regression for (G)EBVs in candidate populations with respect to cC traits. Traits: EW: Egg Weight; ESC: Eggshell Colour; ESS: Eggshell Strength; AH: Albumen Height; ESshape: Eggshell shape index. Candidates: C1: male candidates from G1; C2: male candidates from G1 and G2; F: female candidates from G2. Multi-trait evaluations were performed for the five traits using BLUP (EBV) and ssGBLUP (GEBV) methodologies. Case 1: evaluation at birth; Case 2: evaluation at 60 weeks of age; Case 3: evaluation at 140 weeks of age.

**Additional file 6** Slopes of regression for (G)EBVs in candidate populations with respect to iC traits. Traits: EW: Egg Weight; ESC: Eggshell Colour; ESS: Eggshell Strength; AH: Albumen Height; ESshape: Eggshell shape index. Candidates: C1: male candidates from G1; C2: male candidates from G1 and G2; F: female candidates from G2. Multi-trait evaluations were performed for the five traits using BLUP (EBV) and ssGBLUP (GEBV) methodologies. Case 1: evaluation at birth; Case 2: evaluation at 80 weeks of age; Case 3: evaluation at 160 weeks of age.

## Data Availability

The datasets used and/or analysed throughout the present study are available from the corresponding author upon reasonable request.
